# Infectious Diseases Impact on Biomedical Devices and Materials

**DOI:** 10.1007/s44174-022-00035-y

**Published:** 2022-10-28

**Authors:** Matthew M. Brigmon, Robin L. Brigmon

**Affiliations:** 1 Department of Infectious Diseases and Pulmonary Critical Care, Long School of Medicine, UT Health San Antonio, San Antonio, USA; 2 Savannah River National Laboratory, Bldg 999W, Aiken, SC 29808 USA

**Keywords:** Biomedical Implants, Biofilms, Bacteriophage, Antimicrobial resistance

## Abstract

Infectious diseases and nosocomial infections may play a significant role in healthcare issues associated with biomedical materials and devices. Many current polymer materials employed are inadequate for resisting microbial growth. The increase in microbial antibiotic resistance is also a factor in problematic biomedical implants. In this work, the difficulty in diagnosing biomedical device-related infections is reviewed and how this leads to an increase in microbial antibiotic resistance. A conceptualization of device-related infection pathogenesis and current and future treatments is made. Within this conceptualization, we focus specifically on biofilm formation and the role of host immune and antimicrobial therapies. Using this framework, we describe how current and developing preventative strategies target infectious disease. In light of the significant increase in antimicrobial resistance, we also emphasize the need for parallel development of improved treatment strategies. We also review potential production methods for manufacturing specific nanostructured materials with antimicrobial functionality for implantable devices. Specific examples of both preventative and novel treatments and how they align with the improved care with biomedical devices are described.

## Introduction

Over half of the nearly two million medical care-associated infections can be attributed to indwelling biomedical devices [1]. This accounts for around 25% of all health care-associated infections increases mortality and raises morbidity substantially [2]. Not only the infection but also the rising rates of multidrug-resistant bacteria due to widespread antibiotic use increase the need for multimodality approaches to reduce the infections that require their use [3]. Recent industrial and academic research efforts have focused on the development of nanostructured materials and other novel materials for use in biomedical devices, including medical prostheses, implantable biosensors, and drug delivery devices. Nanostructured materials are defined as materials that contain clusters, crystallites, molecules, or other structural elements with dimensions in the 1 nm–100 nm range [3]. Recent advances in the use of nanostructured materials for medical applications have resulted from two motivations. First, there is a natural evolution to nanoscale materials as novel processing, characterization, and modeling techniques become available. Second, specific interactions between biological structures (e.g., enzymes and other proteins) and nanostructured materials may allow for devices with unusual functionalities to be developed.

## Infections in Medical Devices

The more common infections seen in the medical literature are from foreign materials placed in the brain, vascular catheters, prosthetic cardiac and joint materials, tissue fillers, vascular grafts, breast implants, contact lenses, endotracheal tubes, and urinary catheters causing an array of health concerns (Table 1) [4]. The formation of a favorable bed for infection is complex and it is more common in those patients with altered immune systems and the communication of the device with the environment [1, 5, 6].

**Table 1:** Corresponding device and anatomical infections

Device-related infections	Tissue related infections to device
Ventricular derivations	Cerebral empyema, encephalitis, abscess, otitis media, chronic sinusitis
Contact lenses	Keratitis
Endotracheal tube, tracheostomy	Ventilator associated pneumonia, tracheitis
Vascular central catheters	Septic thrombophlebitis, Endocarditis
Prosthetic cardiac valves, pacemakers, and grafts	Endocarditis, myocardial abscess, and pericardial infections
Peripheral Vascular catheters	Suppurative thrombophlebitis
Tissues fillers, Breast Implants	Cellulitis, abscess
Gastrointestinal stents	Biliary infections
Urinary catheters	Kidney stones, urinary tract infections,
Orthopedic implants and prosthetic joints	Osteomyelitis, septic arthritis, chronic wounds

Depending on the type of organism implicated in the infection and the specific device, removal is usually recommended if feasible. However, eradication of infection has proven to be difficult due to the formation of biofilms. These biofilms start as varying interactions between host-derived proteins that aid in healing post-procedure such as fibronectin, fibrinogen, and vitronectin that encourages the colonization of the surface of implantable devices as observed with *Staphylococcus epidermidis* [7, 8] Once attached, the organisms strengthen their bonds and proliferate and surround themselves with an extracellular polymeric substance (EPS) [9–11]. Microbes will detach from the surface and become active cells causing infections in other sites resulting in more inflammatory responses [12].

Biofilms create aggregation of organisms which are different from the usual planktonic resistance mechanisms [13]. Not only does the presence of EPS alter pH, osmolarity, availability of nutrients, and protection from mechanical forces [14–16], but also blocks the penetration of antibiotics and host immunity [17, 18]. This is further compounded by the fact that slow growth within the biofilm may allow mutations and gene transfer that will contribute to antimicrobial resistance [19–21].

There are many strategies for treating device-related infections. Device removal is usually recommended because there are very few antibiotics that can penetrate biofilms, save some mild evidence for treatment of Methicillin resistant *Staphylococcus aureus* with rifampin [22, 23]. Generally, the antibiotic choice is pathogen-directed along with debridement or removal of the device per guidelines. This also depends on what antibiotic can be given as well as its bioavailability and tissue penetration. Many patients in whom the device cannot be removed will be given lifelong antibiotic suppression. In some cases, this leads to a slow death as the source may be a large vessel or of an endocardial source [24, 25].

## Antimicrobial Resistance

Antibiotic microbial resistance (AMR) is a major concern despite the development of new antimicrobial agents [26]. While antibiotics are considered antimicrobial agents, they may serve as immune modulators as some affect the host immune response [27]. Bloodstream infections (BSIs) attributable to AMR including carbapenem-resistant *Enterobacterales* (CRE-BSIs) are a concern and a major cause of clinical mortality [28]. Patient infections caused by methicillin-resistant *Staphylococcus aureus* (MRSA) result in significant morbidity and mortality in community and clinical settings [26].

The physiological factors that contribute to AMR in biofilms are complex. A study on antibiotic treatment of *Pseudomonas aeruginosa*, a major cystic fibrosis pathogen, demonstrated that it created optimal conditions for the establishment of *Mycobacterium abscessus* infection [29]. The *P. aeruginosa* inhibited *M. abscessus* biofilm formation until antimicrobial therapy selectively targeting *P. aeruginosa* diminished this competitive interaction. Thereby *M. abscessus* flourished, demonstrating the need for careful antimicrobial agent administration.

The US lost progress combating antimicrobial resistance in 2020 due, in large part, to effects of the COVID-19 pandemic [30]. The US Center for Disease Control (CDC) identified significant increases in infections across many healthcare-associated pathogens, such as carbapenem-resistant *Acinetobacter*, extended-spectrum beta-lactamase-producing *Enterobacterales*, vancomycin-resistant *Enterococcus*, and drug-resistant *Candida*. In fact, AMR clinical infections and mortality both increased at least 15% during the first year of the pandemic [30]. This concern for AMR has led to the development of compounds that can serve as antimicrobials for pathogens. This research has included the development of bioactive compounds that work synergistically with polymyxin and demonstrate the potential against AMR bacteria [31].

A major concern with medical and dental biomaterials is colonization with microbial biofilms that patients often cannot eliminate with their own immune response, requiring antimicrobial therapy that can affect the inflammatory response [32]. Host damage, including the immune response to infection, is a concern for patient health. Antimicrobial therapy in consideration of the damage-response framework (DRF) may help tailor-specific therapy to a patient’s need to reduce inflammation[27].

Improved methods to detect AMR are being developed. For example, many US laboratories rely on commercial automated antimicrobial susceptibility tests including minimum inhibitory concentration (MICs), for *Stenotrophomonas maltophilia* that causes lethal infections in immunocompromised patients [33]. The limitations of MIC results for *S. maltophilia* have been documented, so awareness of the limitations of such testing is taken into consideration for treatments.

## Bacteriophages and Other Novel Therapies

Due to AMR, there is emerging need for more effective novel therapies. Recently, bacteriophage therapy has reemerged in the United States and had persisted in the world. In 2020, the National Institute of Allergy and Infectious Diseases (NIAD) and the Antibacterial Resistance Leadership Group (ARLG) created a taskforce regarding the use of phages in clinical practice [34]. This was in response to the growing issue of antimicrobial resistance. They summarized recently published data regarding treatment of organisms such as *Mycobacteria abscessus, Pseudomonas aeruginosa, Burkholderia* spp., *Acinetobacter baumannii, Klebsiella pneumoniae, Staphylococcus epidermidis, Streptococcus agalactiae, Staphylococcus aureus, Cutibacterium acnes*, and *Enterococcus* spp. in a variety of device and non-device-related infections [34]. Suh et al. also reported very few adverse effects associated with bacteriophage therapy. There has been concern that bacteriophages could confer resistance to bacteria, but when given with antibiotics, there is a synergistic effect in lysis [35–38] and will cause bacteria to become more sensitive to other antibiotics[36, 39–41].

The bacteriophages exploit numerous mechanisms of the bacteria and their biofilm. They display a wide variety of different mechanisms in infection including the following: killing active bacteria via direct receptors and gaining access to the biofilm [42–46], some phages encode depolymerases which enable them to degrade biofilm[47–49], exploiting Quorum sensing receptors [50, 51], and inhibiting the ability of fimbria and pili [52]. However, the limitation of the usage of bacteriophages is isolating a specific phage against the bacteria, and this field is still new and requires case by case Food and Drug Administration approval through an Investigational New Drug application as well as Institutional Review Board Approval.

There are also bacteriophages that have been studied as coating for catheters [42, 43] as well as many antibiotic-coated materials for more well-defined targeted therapies [53–55]. Bacteriophages have led to the development of other potential therapies of phage derived lytic proteins that are also known as antimicrobial peptides, which are currently in multiple phases of clinical trials [56, 57].

## Biomedical Materials

New improved materials are being used for specific medical implanted devices to limit infection issues and improve patient outcomes. For example, nanostructured materials may play a significant role in controlled release of pharmacologic agents for the treatment of cancer where immunocompromised patients are of concern [58]. Systemic administration (distribution throughout the entire body) of many common chemotherapeutic agents is associated with significant side effects. For example, the side effects of the common chemotherapeutic agent doxorubicin hydrochloride include myelodysplastic syndrome, congestive heart failure, and mucositis. In addition, many protein- and DNA- based treatments that are being developed for treatment of cancer have relatively short in vivo activities. These chemotherapeutic agents cannot be administered in oral form because they may be metabolized by the liver, intestine, kidneys, or lungs before reaching systemic circulation. Recent work has examined the delivery of chemotherapeutic agents at the site where they are needed; this route avoids diffusional and enzymatic barriers and provides complete and instantaneous absorption. Nanostructured materials may provide constant delivery of a pharmacologic agent to the site in the body where it is needed, providing appropriate treatment over an extended time while minimizing damage to healthy tissue that can enable infection [59].

Nanoporous polymer materials have been found to be inadequate for use in drug delivery. Many porous polymer materials are created using solvent-casting techniques [60]. These materials have poor mechanical properties and large pore size distributions, making precision difficult; for example, pore size variation is as large as 30%. In addition, polymer membranes contain 100–200 µm tortuous pores. Ion-track etching has also been used to form membranes. This technique produces a much narrower pore size distribution than that observed in polymer membranes; for example, pore variation in membranes produced using ion-track etching is within 10%. However, ion-track etched membranes have low porosities; pore concentrations under 10^9^ pores/cm^2^ are commonly observed. Porous silicon is another material that has been considered for use in drug delivery due to its biocompatibility [61, 62]. This material may be produced by electrochemically corroding silicon in solutions containing hydrofluoric (HF) acid. The average diameter of the nanocrystalline porous silicon layers can be modified by altering the electrolyte composition, the electrochemical current, or the dopant chemistry. It should be noted that porous silicon films undergo degradation under physiologic conditions. Although several investigators have examined the use of porous silicon for drug delivery, it is unclear how patient variabilities in physiologic or health status may affect degradation of porous silicon or drug release rates [63].

Nanoporous alumina provides several advantages over other materials for use in controlled drug delivery and other medical applications. It has been shown that that anodization, stripping of the oxide, and re-anodization produce an unusual material with nanoscale pores [64]. Nanoscale pores are randomly formed on the alumina surface at the beginning of the anodization process. These pores self-organize into a hexagonal arrangement during their growth into the bulk material. This first oxide layer is removed using an aqueous solution of 1.8 wt% Cr (VI) oxide and 6 wt% phosphoric acid. A second anodization process is carried out on this template. The resulting material, known as “alumite,” contains long, columnar, ordered nanopores. These nanopores demonstrate long-range order. The structure can be described as close-packed cells in a hexagonal arrangement, with pores at the center of each cell. The pore size can be modified by the selection of appropriate processing temperature, electrical field strength, or electrolyte.

Advances in nanoporous alumina applications have demonstrated their superiority over polymers for use in drug delivery. Alumina is a bioinert ceramic that is stable in physiologic solutions. Finally, the anodization process provides precise control over pore size and pore distribution. However, there is a significant disadvantage to the use of nanoporous alumina materials in medical applications. Although aluminum is a constituent of several medical alloys (e.g., Ti–6Al–4V, ASTM F136), it is currently unknown whether aluminum is a biocompatible material [65]. Nanoporous alumina membrane coated with titanium oxide using atomic layer deposition has proved compatible for biomedical applications [66]. The biocompatibility and resilience of titanium oxide is well established [67]. Titanium oxide is routinely applied as a passivation layer in dental, orthopedic, and cardiovascular implants [68–70]. Having a conformal coating of titanium oxide over the alumina membrane is of importance in order to minimize corrosion and improve cell compatibility. Research has indicated that cells grown on titanium oxide surfaces contaminated by small amounts of alumina exhibit impaired activity; for example, contamination by alumina may lead to impaired mineralization of matrices by osteoblasts (bone cells) [68].

The self-limiting nature of the reaction between these precursors and the surface ensure that all exposed regions of a substrate, including areas that are only accessible via long, tortuous pathways, are coated uniformly and precisely. It is this ability to produce conformal coatings on non-planar substrates that make atomic layer deposition very useful for functionalizing nanoporous materials, including membranes and aerogels [66]. The conformal capability of atomic layer deposition is quite different from that of physical vapor deposition technologies such as evaporation and sputtering, which are limited by line-of-site constraints and can only coat the outer surface of a porous substrate. As such, atomic layer deposition is uniquely suited for depositing a conformal nanometer-scale film with precise thickness onto the surface of a nanoporous membrane.

Atomic layer deposition technology has been developed in recent years for biomedical applications [71]. This technology has been commercialized for a range of applications biosensors. There is currently intense interest in developing atomic layer deposition methods to a range of new applications outside of the realm of microelectronics and biosensors. Much of the ongoing work involves coating nanoporous or nanostructured templates to impart the targeted surfaces with beneficial antimicrobial functionalities [66]. For example, nanoporous alumina membranes are a convenient platform for synthesizing nanotubes and nanowires using atomic layer deposition-based templating methods for biosensor development.

For nanoparticles to have optimal long-term antimicrobial activity, they are typically attached to solid surfaces in procedures designed to prepare heterogeneous composites for specific applications [71]. Combining materials can have a synergistic effect as it was demonstrated that TiO_2_/ZnO nanoparticles supported in 4A zeolite could lead to resilient activity as antimicrobial agents [72]. In fact, multilayered composite coatings of TiO_2_ nanotubes combined with ZnO and hydroxyapatite nanoparticles were applied for controlled Zn release for antimicrobial activity against *Staphylococcus aureus* [73]. Biological compatibility of TiO_2_ coatings by changing the surface chemical composition and nanotopography has supported its use in biomedical TiO_2_ applications [74]. However, application of nanomaterials including TiO_2_ is a concern due to the potential ecotoxicological effects. They have been found to inhibit the growth of algae [75] and fish [76]. Therefore, in manufacturing or use, care must be taken to assure the coatings or application must be resilient and durable. Earlier implant Ti alloy applications had possible toxic effects resulting from released vanadium (V) and aluminum (Al). For this reason, vanadium- and aluminum-free alloys have been introduced for biomedical applications [77].

Nanostructured materials prepared using atomic layer deposition may be useful for delivering a pharmacologic agent at a precise rate to a specific location of the body. Ceramic materials are associated with less inflammation than polymeric materials that are currently used for local drug delivery. In addition, recent advances in nanoporous ceramics may provide greater control over release rate than polymers, since there are often difficulties when pharmacologic agents and polymers are dissolved in a given solvent [78]. These materials may serve as the basis for “intelligent” drug delivery, which allows for controlled release of a pharmacologic agent in response to electric field, magnetic field, pH, temperature, or light intensity. An intelligent system could release a gene or drug at a precise rate to the location in the body where it is needed to limit infections and increase effectiveness. Nanoporous titanium coatings may also be useful for improving bone synthesis and associated tissue growth in orthopedic implants or in preventing infection [79, 80]. In summary, the analysis of risk factors and AMR agent and biomaterial efficacies has the potential to guide the management of these patients. However, future prospective cohort studies or randomized trials are urgently needed to validate and expand upon the findings of the current review.

## Conclusions

The number of implantable devices will only continue to expand as therapies become more widely available and applicable in an ever-growing population. The importance of lower the mortality and morbidity of these devices as well as their treatment is a race to reduce cost and increase the lifetime of the materials. There is still much to learn with regard to biomaterials, the host’s response to infection and material, and the microbial interactions with all of the above. A combined multi-modal approach in including targeted therapeutics seems to be the best approach. Hopefully, we will be able to develop materials and antimicrobial therapies that can maintain effective treatment for current and emerging infectious agents.
